# Plectin in Health and Disease

**DOI:** 10.3390/cells11091412

**Published:** 2022-04-21

**Authors:** Gerhard Wiche

**Affiliations:** Max Perutz Laboratories, Department of Biochemistry and Cell Biology, University of Vienna, 1030 Vienna, Austria; gerhard.wiche@univie.ac.at

In light of recent progress in defining a unifying mechanism for the versatile functions and disease involvement of the cytolinker protein plectin, a series of invited review articles, together with an original research article, were published in Cells as a Special Issue entitled ‘Plectin in Health and Disease’.

Since its molecular identification some forty years ago, plectin emerged as a central player in the organization and performance of the vertebrate cell cytoskeleton. It has been recognized as an essential mediator of intermediate filament (IF) network interactions, with impact on the spatial compartmentalization and modulation of the actomyosin machinery as well as on microtubule network dynamics. Due to its isoform diversity based on alternative first exon splicing and its high-affinity binding to IFs independent of their type, plectin acts as a universal recruiter and anchoring platform of cytoplasmic IF networks. Specific anchorage occurs at all sorts of cytoskeletal junctional complexes (among others, hemidesmosomes, focal adhesions, desmosomes, adherens and β-dystroglycan junctions, the neuromuscular synapse, and invadopodia of metastatic cancer cells), as well as at the cytoplasmic face of organelles (in particular mitochondria and the nuclear/ER membrane). In addition, plectin directly couples IFs to the actomyosin machinery with major implications on cytoarchitecture, polarization, migration potential, and mechanotransduction of cells. 

Based on plectin’s broad interaction potential, strategic locations, and ample functional repertoire, dysfunctional plectin leads to a variety of disorders and diseases. In fact, plectin can be considered as a paradigm of a multisystemic disease-causing gene, where different tissues, cell types, and organs, are phenotypically affected in humans and mouse animal models. The type of human diseases caused by plectin loss or dysfunction, and the phenotypes of the corresponding animal models, clearly indicate that plectin plays an important role in maintaining the structural and functional integrity of cells and tissues exposed to great mechanical stress, such as the skin, muscle, intestine, and vasculature. In accordance with its essential role in mechanotransduction and cell motility, plectin also features prominently in metastatic cancer cells. 

The key studies that led from plectin’s roots to its recognition and establishment as a universal recruiter and essential networking element of IFs are highlighted in [1]. The role of plectin and its specific isoforms in IF network organization and multitasking is viewed from different perspectives, in particular: (i) mechanical network and docking site stabilization; (ii) contribution to cellular viscoelasticity; (iii) signaling and mechanotransduction; (iv) compartmentalization and control of the actomyosin machinery; (v) crosstalk with the microtubule system; and (vi) plectin and IFs being equal and mutually dependent partners. The article culminates in the presentation of a new working model for a unifying mechanism underlying the control and propagation of actomyosin-generated forces through plectin–IF networks, regulation of microtubule dynamics, and mechanotransduction involving diverse peripheral junctional complexes, mitochondria, and the nucleus.

The initial analyses of plectin’s molecular properties performed in vitro and ex vivo have been validated in vivo by studying a plethora of mouse models and the clinical and histological phenotypes of patients carrying plectin mutations. As shown in [2], a great part of what is known to date about the biological functions and disease mechanisms of plectin has been gained through the analysis of well over a dozen different transgenic mouse models. The complex pattern of alternative plectin gene splicing and tissue/cell type-specific distribution of transcripts required the creation and analyses of a variety of isoform-specific, tissue- and cell type-specific (conditional), and double knockout mouse lines, as well as knock-in lines, aside from full knockouts. This approach enabled the dissection of isoform-specific functions in a tissue/cell type-dependent manner. The systems that were studied in this way comprise muscle satellite cells, skeletal muscle and heart, skin, vascular endothelial cells, neurons, Schwann cells, liver, small intestine, myofibers, keratinocytes, fibroblasts, leukocytes, kidney cells, and others. The article summarizes the key molecular features and pathological phenotypes that were identified in these various organisms and their derivative cell systems, and evaluates their aptitude as model systems for human plectinopathies. Finally, it highlights the work carried out with C. elegans, which proved as an alternative powerful animal model system to study functional characteristics of plectin on the molecular level. 

The involvement of plectin in disease became known some 25 years ago when the first patients suffering from epidermolysis bullosa simplex with muscular dystrophy (EBS-MD) were shown to be carriers of plectin mutations. EBS-MD, which turned out to be the most common plectinopathy variant, phenotypically manifests with skin blistering and muscular disorders. In fact, skin blistering is the predominant early-on hallmark of most plectinopathies (as well as of plectin-deficient mice), while muscular disorders usually develop at later stages. This situation is described in detail in two reviews, each one focusing on a particular tissue. In skin, plectin functions as a mediator of keratinocyte mechanical stability mainly through linking of the intermediate filament network to hemidesmosomes, with concomitant suppression of counteracting contractile actomyosin forces. The cutaneous manifestations of plectinopathies as well as their systemic involvement in the specific disease subtypes are summarized by Kiritsi et al. [3]. This is an important topic considering the phenotypic variability of plectin-related dermatoses. The article deals also with plectin’s involvement in autoimmune blistering disorders, bullous pemphigoid and specifically paraneoplastic pemphigus (PNP). The pathogenicity and development of anti-plectin antibodies in PNP (as with that of other PNP antigens) remain enigmatic, and so does the cell surface exposure of plectin (CSP) in metastatic cancer cells (see Special Issue articles by Perez et al). Thus, it will be intriguing to find out whether similar mechanisms are involved in both phenomena. 

The multiple functions of plectin in muscle are reviewed in the Special Issue article on muscle-related plectinopathies, where Zrelski et al. [4] address the clinical and pathological manifestations caused by *PLEC* mutations in skeletal and cardiac muscle. The article systematically covers a broad spectrum of studies with skeletal muscle biopsies from EBS-MD patients as well as genetically altered mice. Specimens of both types revealed severe dystrophic features, such as variation in fiber size, degenerative myofibrillar changes, mitochondrial alterations and pathological desmin-positive protein aggregates. Ultrastructurally, *PLEC* mutations lead to a disorganization of myofibrils and sarcomeres, Z- and I-band alterations, autophagic vacuoles and cytoplasmic bodies, along with misplaced and degenerating mitochondria. A substantial part of the review is devoted to diverse genetically manipulated mouse and cell models, which are either plectin-deficient or specifically lack a skeletal muscle-expressed plectin isoform. These models proved to be powerful tools in studying functional and molecular consequences of *PLEC* defects and their downstream effects on the skeletal muscle organization. Moreover, they proved suitable for screening and testing of pharmacological compounds for future therapeutic approaches.

In spite of plectin having been found early on to be abundantly expressed in the central and peripheral nervous systems (C/PNS), and some of the patients with EBS-MD having been diagnosed with CNS pathology, studies on plectin’s role in neural cells have been scarce. As reviewed by Potokar and Jorgačevski [5] in this Special Issue series, apart from a couple of studies on plectin in neurons and Schwann cells from isoform and conditional plectin knock out mice, plectin’s functions in brain and the nervous system have remained largely unexplored. This applies especially to isoform-specific functions in cells of the CNS where high-level expression of plectin occurs, such as in astrocytes, ependymal cells, oligodendrocytes, and Bergmann glia. In addition to summarizing current knowledge, the review focusses on cellular processes in which plectin is expected to play an important role, based on insights gained from studies on non-neural cell systems. This includes the whole spectrum of plectin-mediated mechanical and signaling functions of IFs. The assessment of whether the variability in expression patterns of IFs coupled with specific isoforms of plectin is linked to developmental and pathological stages of individual cell types, and the potential role of plectin and its isoforms as biomarker of CNS neoplasms, are considered key challenges for future studies in the neuroscience field. 

One of the most exciting developments regarding plectin-related research in recent years was the emergence of plectin as a potent driver of malignant hallmarks in many human cancers. As shown in the Special Issue review article by Perez et al. [6], this newly identified feature is based on plectin’s involvement in various cellular activities contributing to tumorigenesis, including cancer cell proliferation, adhesion, migration, invasion, and signal transduction. To quote the authors: “Evidence shows that beyond plectin’s diverse protein interactome, its cancer-specific mislocalization to the cell surface enables its function as a potent oncoprotein. As such, therapeutic targeting of plectin, its protein interactors, and in particular, cancer-specific plectin (CSP), presents an attractive opportunity to impede carcinogenesis directly”. The article reports on plectin’s differential gene and protein expression in cancer, explores its mutational profile, and discusses the current understanding of plectin’s and CSP’s biological function in cancer. Moreover, the prospects of plectin as a prognostic marker, diagnostic biomarker, and target for imaging and therapeutic modalities is rigorously reviewed. It is concluded that plectin’s common overexpression in cancer and CSP’s cancer-specific bioavailability bears potential as high-value druggable targets. In fact, the same group published an original research article in this Special Issue series, where first solid evidence for a potent anticancer effect of CSP therapeutic targeting is presented. In their study, Perez et al. [7] developed a first-in-class anti-CSP monoclonal antibody (mAb 1H11), and tested it alone (monotherapy) and in combination with standard of care chemotherapies to treat ovarian cancer. Strikingly, mAb 1H11 showed robust anticancer effects as a single agent in in vitro and in vivo ovarian cancer models. Moreover, mAb 1H11 synergized with the first-line therapy agent cisplatin and dual treatment caused a sustained reduction in tumor growth compared to cisplatin alone. Revealing mAb 1H11’s utility for integration with current mainstay drugs has wide implications on future combinatorial treatment strategies in ovarian cancer. This study not only opens the door for further avenues of investigation into CSP, but its cancer-specific expression, and functional significance also demonstrate the potential of other noncanonical cell surface proteins to be exploited for anticancer interventions.

Since the publication of this Special Issue, a couple of research articles came out that have strong bearing on some of the addressed topics. For instance, the spectrum of cytoskeletal junctions requiring plectin-mediated orchestration of cortical IF and actomyosin networks for proper performance can meanwhile be expanded to intercellular adherens junctions and desmosomes of epithelial sheets based on the report by Prechova et al. [8]. Their study, showing that defective cytoarchitecture and tensional disequilibrium due to plectin deficiency leads to reduced intercellular cohesion, associated with general destabilization of epithelial cell sheets upon mechanical stress, has high physiological relevance (see also [9]). Along these lines, plectin-mediated IF-actomyosin filament crosstalk was shown to affect autophagosome-lysosome fusion efficiency during macroautophagy under oxidative stress condition [10].

In conclusion, composed of six specialized reviews and one research article, this Special Issue presents an updated overview on plectin-related research, providing new mechanistic insights into plectin’s global role as mediator of cytoskeleton crosstalk and dynamics, and describing its role in disease and emergence as tumor marker and therapeutic target of metastatic cancer cells. We hope that these articles stimulate further research into not yet thoroughly explored territories, such as the nervous system and cartilageous tissues, and inspire the scientific community to develop efficient therapeutic interventions for the multiple plectin-related diseases.
